# European recommendations for short-term surveillance of health problems in childhood, adolescent and young adult cancer survivors from the end of treatment to 5 years after diagnosis: a PanCare guideline

**DOI:** 10.1007/s11764-023-01493-z

**Published:** 2023-12-04

**Authors:** Ismay A. E. de Beijer, Roderick Skinner, Riccardo Haupt, Desiree Grabow, Edit Bardi, Andrea Beccaria, Adela Cañete Nieto, Samira Essiaf, Anna-Liesa Filbert, Hannah Gsell, Anita Kienesberger, Thorsten Langer, Patricia McColgan, Monica Muraca, Jelena Rascon, Ramona Tallone, Zuzana Tomasikova, Anne Uyttebroeck, Leontien C. M. Kremer, Helena J. H. van der Pal, Renée L. Mulder, on behalf of the PanCareSurPass consortium, Desiree Grabow, Anna-Liesa Filbert, Dorothea Niehoff, Diana Walz, Friederike Erdmann, Claudia Spix, Riccardo Haupt, Monica Muraca, Simone Lightwood, Francesca Bagnasco, Giacomo Cavalca, Sara Oberti, Brigitte Nicolas, Ruth Ladenstein, Edit Bardi, Vanessa Düster, Anne Uyttebroeck, Maria van Helvoirt, Jurgen Lemiere, Marleen Renard, An Michiels, Thorsten Langer, Ann-Kristin Kock-Schoppenhauer, Lea Hildebrand, Anke Neumann Anne-Katrin Jahnke, Jelena Rascon, Justas Trinkūnas, Audronė Ciesiūnienė, Paulius Ragauskas, Adela Cañete Nieto, Julia Balaguer Guill, Maria Teresa Tormo Alcañiz, Antonio Orduña Galan, Marisa Correcher Palau, Lucas Cervero Beltrán, Vicente Pons Tamarit, Davide Saraceno, Alessandra Berti, Carlo Contino, Nikos Thomopulos, Giulia Stabile, Maria Franca Tomassi, Igor Zamberlan, Barbara Nichel, Günter Schreier, Dieter Hayn, Karl Kreiner, Stefan Beyer, Catherine Chronaki, Giorgio Cangioli, Eliana Charalambous, Alexander Degelsegger-Márquez, Gerald Gredinger, Kathrin Trunner, Florian Trauner, Anja Laschkolnig, Leontien Kremer, Heleen van der Pal, Saskia Pluijm, Selina van den Oever, Ismay de Beijer, Jessica Trollip, Emma Hardijzer, Heleen van der Pal, Jaap den Hartogh, Jeroen te Dorsthorst, Samira Essiaf, William Sciberras, Anita Kienesberger, Hannah Gsell, Carina Schneider, Zuzana Tomasikova

**Affiliations:** 1 Princess Maxima Center for Pediatric Oncology, Heidelberglaan 25, 3584 CS Utrecht, The Netherlands; 2 Wolfson Childhood Cancer Research Centre, Newcastle University Centre for Cancer, Herschel Building, Brewery Lane, Newcastle Upon Tyne, NE1 7RU UK; 3 Great North Children’s Hospital, Royal Victoria Infirmary, Queen Victoria Road, Newcastle Upon Tyne, NE1 4 LP UK; 4 Translational and Clinical Research Institute, Wolfson Childhood Cancer Research Centre, Herschel Building, Brewery Lane, Newcastle Upon Tyne, NE1 7RU UK; 5 IRCCS Istituto Giannina Gaslini, Genoa, Italy; 6 Division of Childhood Cancer Epidemiology/German Childhood Cancer Registry, Institute of Medical Biostatistics, Epidemiology and Informatics (IMBEI), University Medical Centre of the Johannes Gutenberg University Mainz, Mainz, Germany; 7 St. Anna Children’s Hospital, Vienna, Austria; 8 Department of Paediatrics and Adolescent Medicine, Johannes Kepler University Linz, Linz, Austria; 9 Hospital Universitario y Politécnico La Fe, Valencia, Spain; 10 European Society for Paediatric Oncology, C/O BLSI, Clos Chapelle-Aux-Champs 30, Bte 1.30.30, Brussels, Belgium; 11 CCI Europe, Vienna, Austria; 12 Universitatsklinikum Schleswig-Holstein, Campus Lubeck, Lubeck, Germany; 13 Childhood Cancer Ireland, Carmichael House, 4 Brunswick Street North, Dublin, D07 RHA8 Ireland; 14 Vilnius University Hospital Santaros Klinikos, Vilnius, Lithuania; 15 University Hospitals Leuven, KU Leuven, Louvain, Belgium; 16 University Medical Center Utrecht, Wilhelmina Children’s Hospital, Utrecht, The Netherlands; 17 Department of Pediatrics, Emma Children’s Hospital, Amsterdam UMC, University of Amsterdam, Amsterdam, The Netherlands

**Keywords:** Paediatric oncology, Short-term follow-up care, Survivorship, Aftercare, Practice guideline, Cancer survivors, Quality of life, Survivorship Passport, Surveillance

## Abstract

**Purpose:**

Childhood, adolescent and young adult (CAYA) cancer survivors require ongoing surveillance for health problems from the end of cancer treatment throughout their lives. There is a lack of evidence-based guidelines on optimal surveillance strategies for the period from the end of treatment to 5 years after diagnosis. We aimed to address this gap by developing recommendations for short-term surveillance of health problems based on existing long-term follow-up (LTFU) care guidelines.

**Methods:**

The guideline working group, consisting of healthcare professionals, parents and survivor representatives from 10 countries, worked together to identify relevant health problems that may occur in survivors between the end of treatment and 5 years after diagnosis and to develop recommendations for short-term surveillance of health problems. The recommendations were drawn from existing LTFU guidelines and adapted where necessary based on clinical expertise.

**Results:**

The working group developed 44 recommendations for short-term surveillance of health problems, which were divided into four categories based on the level of surveillance required: awareness only (*n* = 11), awareness, history and/or physical examination without surveillance test (*n* = 15), awareness, history and/or physical examination with potential surveillance test (*n* = 1) and awareness, history and/or physical examination with surveillance test (*n* = 17).

**Conclusion:**

The development of a guideline for short-term surveillance of health problems fills a critical gap in survivorship care for CAYA cancer survivors, providing much-needed support immediately after treatment up to 5 years after diagnosis.

Implications for Cancer Survivors.

This guideline will support healthcare professionals to provide appropriate follow-up care and improve the quality of life of CAYA cancer survivors.

**Supplementary information:**

The online version contains supplementary material available at 10.1007/s11764-023-01493-z.

## Introduction

Worldwide, approximately 400,000 children and adolescents aged 18 years and younger are diagnosed with cancer each year [1]. Because of improved cancer treatments, there are currently around 500,000 childhood, adolescent and young adult (CAYA) cancer survivors living in Europe [2, 3]. However, due to the adverse effects of cancer treatment, CAYA cancer survivors are at high risk of developing health problems, including a wide range of physical and psychosocial conditions such as chronic pain, cardiomyopathy and impaired fertility [4–15]. Appropriate follow-up care is essential to mitigate these health problems, maintain health and preserve quality of life for survivors and their families [16–18].

High-quality follow-up care for CAYA cancer survivors is based on evidence-based guidelines. For long-term follow-up (LTFU) care (survivorship care more than 5 years after CAYA cancer diagnosis), guidelines for surveillance of late health problems have been developed and harmonised in Europe by the Pan-European Network for Care of Survivors after Childhood and Adolescent Cancer (PanCare) in the ongoing PanCareFollowUp project (www.pancarefollowup.eu) and globally by the International Late Effects of Childhood Cancer Guideline Harmonization Group (IGHG) with input from the Children’s Oncology Group (COG), Dutch Childhood Oncology Group (DCOG), Scottish Intercollegiate Guidelines Network (SIGN) and the UK Children’s Cancer and Leukaemia Group (UKCCLG) [19–38]. However, while CAYA cancer survivors need a continuum of survivorship care from the end of their cancer treatment throughout their lives, guidelines for short-term surveillance of health problems—the period from the end of treatment to 5 years after diagnosis—are currently lacking.

To address this gap, the European PanCareSurPass project (www.pancaresurpass.eu, Project Grant Agreement 899999) aimed to develop recommendations for short-term surveillance of health problems in CAYA cancer survivors. These recommendations for short-term surveillance of health problems will be relevant to the care of CAYA survivors from the end of treatment to 5 years after diagnosis. In addition, they can be implemented in the PanCare Survivorship Passport (SurPass) in Europe. The SurPass is a digital tool that contains the treatment summary of patients who have completed cancer therapy and provides a personalised survivorship care plan for late health problems and follow-up care based on the IGHG and PanCare guidelines [39]. This paper describes the development and results of the European harmonised guideline for short-term surveillance of health problems, including recommendations for surveillance of health problems and health promotion in CAYA survivors from the end of treatment to 5 years after diagnosis.

## Methods

### Guideline working group

A guideline working group was formed to develop the PanCare guideline for short-term surveillance of health problems. The working group consisted of 19 stakeholders (survivorship care specialists, researchers and survivor representatives) from 10 European countries. A core group of six people designed the methodology and guided the development of the recommendations.

### Guideline methodology

The guideline working group first reviewed the PanCare guideline on LTFU care for CAYA cancer survivors [38]. This LTFU guideline incorporated 16 evidence-based global IGHG guidelines published up to 2020, as well as consensus-based recommendations for health problems for which there were no evidence-based IGHG guidelines. The working group then reviewed the newly published evidence-based IGHG guidelines from 2021 onwards [27, 29–37].

The group compiled a list of all relevant recommendations for health problems requiring surveillance strategies for the period from the end of treatment to 5 years after diagnosis. For each health problem, we considered the following key questions: (1) Who is at risk? (2) Which surveillance test should be used? (3) When should surveillance be initiated? (4) At what frequency should surveillance be performed? (5) What should be done when abnormalities are found? For health problems considered relevant, we adopted the IGHG guidelines [14–32]. Where IGHG efforts were still ongoing or not yet initiated, we used the consensus-based surveillance recommendations from the PanCare LTFU care guideline as a starting point [33]. For each topic of the LTFU care guideline, we determined whether the existing recommendations covered the period from the end of treatment to 5 years after diagnosis. If so, we adopted the recommendations. If not, we discussed the recommendations in the working group and adapted them based on clinical expertise. Specifically, we adapted the recommendations for short-term surveillance of health problems care with regard to the time of initiation and frequency of surveillance.

### Internal and external consultation rounds

We organised five online meetings to discuss the draft recommendations within the working group. Each working group meeting covered approximately 5–6 health problems, and working group members were invited to comment on the draft recommendations in advance. After the working group meetings, we circulated a full draft of the recommendations to the PanCareSurPass Consortium and the PanCare Guideline Group for feedback. We collected feedback on the draft during a 2-week consultation period. A final online meeting was held to discuss the feedback received and to reach consensus on the wording of the recommendations. We made the necessary revisions to the recommendations based on the feedback received, resulting in the final version of the PanCare guideline on short-term surveillance of health problems. We circulated this final version for approval to the PanCareSurPass Consortium, the PanCare Guideline Group (a separate PanCare committee that promotes and supports the development and implementation of guidelines for CAYA cancer survivors in Europe https://www.pancare.eu/for-professionals/guidelines/) and the PanCare 2022 Board. The entire guideline development process lasted from April 2022 to August 2023.

## Results

### Overview of the PanCare guideline for short-term surveillance of health problems

The PanCare guideline for short-term surveillance of health problems for CAYA cancer survivors includes a total of 44 health problems. The guideline is organised according to the type of counselling or surveillance required, with 11 requiring “Awareness only”, 15 requiring “Awareness, history and/or physical examination without surveillance test”, 1 requiring “Awareness, history and/or physical examination with potential surveillance test” and 17 requiring “Awareness, history and/or physical examination with surveillance test”. The full guideline and the changes made from the LTFU care guidelines are documented in Online Resource 2. Table 1 provides a comprehensive overview of the recommendations for short-term surveillance that include surveillance testing.

**Table 1:** Overview of recommendations for short-term surveillance for health problems involving surveillance tests

Recommendation for short-term surveillance of:	Who is at risk? CAYA cancer survivors treated with or with a history of …	What surveillance test should be used, when should it be initiated and at what frequency?*
Cardiac problems (arrhythmia) Consensus-based PanCare recommendations [38]	• Radiotherapy ≥ 15 Gy to a volume exposing the heart • Anthracyclines, including doxorubicin, daunorubicin, epirubicin, idarubicin and mitoxantrone	• ECG once at the end of treatment • Repeat ECG once after the age of 18 years, if the end of treatment was at a younger age
Cardiac problems (cardiomyopathy) Evidence-based IGHG recommendations [21]	• Radiotherapy to a volume exposing the heart • Anthracyclines, including doxorubicin, daunorubicin, epirubicin, idarubicin and mitoxantrone	• Echocardiogram with assessment of left ventricular systolic function: ▪ Radiotherapy ≥ 30 Gy to a volume exposing the heart: twice every 5 years, starting no later than 2 years after cardiotoxic therapy ▪ Total cumulative anthracycline dose ≥ 100–250 mg/m^2a^: every 5 years, starting no later than 2 years after cardiotoxic therapy ▪ Total cumulative anthracycline dose ≥ 250 mg/m^2a^: twice every 5 years, starting no later than 2 years after cardiotoxic therapy ▪ Combination of radiotherapy ≥ 15 Gy to a volume exposing the heart and total cumulative anthracycline dose ≥ 100 mg/m^2a^: twice every 5 years, starting no later than 2 years after cardiotoxic therapy • Anthracyclines, mitoxantrone and/or radiotherapy to a volume exposing the heart: prior to pregnancy or in the first trimester. Continuing cardiomyopathy surveillance is reasonable during pregnancy for female survivors treated with anthracyclines or chest RT who had a history of prior LV systolic dysfunction that has resolved even in the presence of a normal baseline ejection fraction in the first trimester
Cardiac problems (pericardial and valvular heart disease) Consensus-based PanCare recommendations [38]	• Radiotherapy ≥ 15 Gy to a volume exposing the heart	• Echocardiogram with specific attention to the pericardium and valvular structure and function: at least every 5 years, starting no later than 2 years after cardiotoxic therapy
CNS neoplasms (meningiomas, (high-grade) gliomas and other CNS neoplasms) Evidence-based IGHG recommendations [27]	• Radiotherapy to a volume exposing the head or brain, including TBI	• No recommendation can be formulated for routine MRI surveillance for asymptomatic survivors. The decision to undertake MRI surveillance should be made by the CAYA cancer survivor and HCP after careful consideration of the potential harms and benefits of MRI surveillance
Ear problems (hearing loss and tinnitus) Evidence-based IGHG recommendations [25]	• Cisplatin (with or without carboplatin > 1500 mg/m^2^) • Radiotherapy ≥ 30 Gy to a volume exposing the head or brain	Survivors < 6 years of age at risk: • Extensive testing by audiologist every year, to begin no later than the end of treatment Survivors ≥ 6 years of age at risk • Pure tone conventional audiometry testing at 1000–8000 Hz • Additional testing with high-frequency audiometry > 8000 Hz (whenever equipment is available), to begin no later than the end of treatment; every other year if 6–12 years of age, every 5 years for adolescents and young adults ≥ 12 years of age
HP axis problems (GHD, TSHD, LH/FSHD and ACTHD) Evidence-based IGHG recommendations [37]	• Radiotherapy to a volume exposing the HP region, including TBI (if ≥ 30 Gy, refer directly to (paediatric) endocrinologist or see in multidisciplinary team) • Surgery near or within the HP region (refer directly to (paediatric) endocrinologist or see in multidisciplinary team) • A CNS tumour near or within the HP region (refer directly to (paediatric) endocrinologist or see in multidisciplinary team) • Hydrocephalus or cerebrospinal fluid shunt (at risk for GHD)	Pre-pubertal and peri-pubertal survivors at risk: • fT4, TSH, morning cortisol every year, starting at ≥ 1 year after completion of radiotherapy or directly after hydrocephalus or CSF shunt occurrence Post-pubertal survivors at risk: • fT4, TSH, morning cortisol, IGF-1 • Morning testosterone, or free testosterone if overweight and LH (males) • Estradiol, FSH and LH (females) every year starting at ≥ 1 year after radiotherapy or directly after hydrocephalus or CSF shunt occurrence Note: an IGF-1 level even as high as 0 SDS does not rule out GHD Note: continue surveillance at least 15 years from exposure. Continuation of surveillance should be a shared decision between survivor and HCP considering available health care resources. If surveillance is terminated, the survivor should be educated about possible signs and symptoms of HP axis problems
Impaired glucose metabolism and diabetes mellitus Consensus-based PanCare recommendations [38]	• Radiotherapy to a volume exposing the pancreas, including TBI	• Fasting blood glucose with or without HbA1c at least every 5 years starting at end of treatment
Iron overload Evidence-based IGHG recommendations [36]	• HSCT • Multiple red blood cell transfusions	• Serum ferritin once at end of treatment
Late liver injury (liver fibrosis or cirrhosis, hepatocellular liver injury, hepatobiliary dysfunction, biliary tract injury or liver synthetic dysfunction) Evidence-based IGHG recommendations [36]	• Radiotherapy to a volume exposing the liver, including TBI • HSCT • Methotrexate • Mercaptopurine • Thioguanine • Dactinomycin • Busulfan • Sinusoidal obstruction syndrome • cGvHD • Liver surgery • Chronic viral hepatitis (follow-up by appropriate specialist, e.g. hepatologist or infectious disease specialist, according to local or national hepatitis CPGs)	• Serum liver enzyme concentrations (ALT, AST, gGT, ALP) once at the end of treatment
Male fertility problems and sexual dysfunction (impaired fertility, impaired spermatogenesis, testosterone deficiency and physical sexual dysfunction) Evidence-based IGHG recommendations [33]	• Alkylating agents • Radiotherapy to a volume exposing the testes, including TBI • Surgery to the spinal cord, sympathetic nerves or pelvis • Hypogonadism	Post-pubertal survivors treated with radiotherapy ≥ 12 Gy to a volume exposing the testes, including TBI: • Early morning testosterone at clinically appropriate time intervals • LH in addition to (early morning) testosterone if clinical signs of hypogonadism, previous low or borderline testosterone concentrations, or if an early morning testosterone sample cannot be obtained, at least every 2–3 years Post-pubertal survivors at risk that desire assessment of potential for future fertility: • Semen analysis
Overweight and obesity Consensus-based PanCare recommendations [38]	• Hypothalamic or pituitary tumour • Radiotherapy to a volume exposing the hypothalamus or pituitary gland, including TBI • Neurosurgery of hypothalamus or pituitary gland	• Height, weight and BMI at least every 2 years and at every follow-up visit
Precocious puberty (central) Evidence-based IGHG recommendations [37]	• Radiotherapy to a volume exposing the HP region, including TBI (if ≥ 30 Gy, refer directly to (paediatric) endocrinologist or see in multidisciplinary team) • Surgery near or within the HP region (refer directly to (paediatric) endocrinologist or see in multidisciplinary team) • A CNS tumour near or within the HP region (refer directly to (paediatric) endocrinologist or see in multidisciplinary team) • Hydrocephalus or cerebrospinal fluid shunt	All survivors at risk: • Height velocity in relation to parental height • Tanner stage: every 6 months starting at ≥ 1 year after completion of radiotherapy or directly after hydrocephalus or CSF shunt occurrence Note: Continue surveillance until the age of 8 years for girls and 9 years for boys. Boys exposed to radiotherapy to the testes may have testes small for pubertal stage while in puberty. Instead, morning testosterone (before 10:00 a.m.) should be used as screening modality as testicular volume may be unreliable
Premature ovarian insufficiency (impaired fertility, amenorrhoea and premature menopause) Evidence-based IGHG recommendations [22]	• Alkylating agents • Radiotherapy to a volume exposing the ovaries, including TBI	Pre- and peri-pubertal survivors at risk: • FSH and oestradiol^b^ in case of failure to initiate or progress through puberty at least for girls ≥ 11 years of age, and for girls with primary amenorrhoea (16 years of age) Post-pubertal survivors at risk: • FSH and oestradiol^b,c^ in case of menstrual cycle dysfunction suggesting premature ovarian insufficiency, or if assessment of potential for future fertility is desired
Pulmonary problems (pulmonary dysfunction and worsening pulmonary fibrosis after high oxygen exposure in survivors treated with bleomycin who already have evidence of pulmonary fibrosis) Consensus-based PanCare recommendations [38]	• Carmustine (BCNU) • Lomustine (CCNU) • Busulfan • Bleomycin • Radiotherapy to a volume exposing the lungs, including TBI • Allogeneic HSCT • Thoracic surgery	• Pulmonary function tests, including spirometry and diffusing capacity for carbon monoxide (DLCO), once at the end of treatment or at the age of 6 years, whichever occurs last
Reduced bone mineral density Evidence-based IGHG recommendations [35]	• Cranial or craniospinal radiotherapy • TBI • Corticosteroids as anti-cancer treatment, at least 4 weeks continuously	Survivors treated with cranial or craniospinal radiotherapy or TBI: • A DXA scan once, 2–5 years after the end of treatment, and thereafter as clinically indicated Note: It might be considered to postpone the DXA-scan in pre-pubertal and pubertal survivors Survivors treated with corticosteroids as anti-cancer treatment: • No recommendation can be formulated for or against BMD surveillance; the decision to undertake surveillance should be made together by the survivor and healthcare provider, after careful consideration of the potential harms and benefits
Renal problems (glomerular and tubular dysfunction) Consensus-based PanCare recommendations [38]	• Ifosfamide • Cisplatin • Carboplatin • Radiotherapy to a volume exposing the kidney or urinary tract, including TBI • Nephrectomy • HSCT	All survivors at risk: • Glomerular function testing including blood testing (creatinine), urine testing (creatinine, proteinuria), eGFR calculation at least every 5 years starting at the end of treatment Survivors treated with ifosfamide, cisplatin or carboplatin: • Additional tubular function testing including blood testing (Na, K, Mg, P, Ca, phosphate, albumin) and urine testing (glucose, phosphate) at least every 5 years starting at the end of treatment
Thyroid function problems (hypothyroidism and hyperthyroidism^d^) Consensus-based PanCare recommendations [38]	• Radiotherapy to a volume exposing the thyroid gland, including TBI • Radioiodine therapy (I-131 ablation therapy) • MIBG therapy (I-131 MIBG therapy)^e^ • Allogeneic HSCT • Total thyroidectomy (follow-up by an endocrinologist starting directly after surgery)	Survivors treated with radiotherapy or allogeneic HSCT: • TSH and fT4 measurement every year in survivors ≤ 18 years of age and at least every 2–3 years in survivors > 18 years of age Survivors treated with radioiodine/MIBG therapy: • TSH and fT4 measurement 1, 3, 6 and 12 months after radioiodine/MIBG therapy and afterwards every year in survivors ≤ 18 years of age, and at least every 2 years in survivors > 18 years of age Female survivors at risk of hypothyroidism: • TSH and fT4 prior to attempting pregnancy and periodically during pregnancy

*ACTHD* adrenocorticotropic hormone deficiency, *ALP* alkaline phosphatase, *ALT* alanine aminotransferase,* AST* aspartate aminotransferase, *AML* acute myeloid leukaemia, *CAYA* childhood, adolescent and young adult, *cGvHD* chronic graft-versus-host disease, *CNS* central nervous system, *CPG* clinical practice guideline, *dB* decibel, *DXA* dual-energy X-ray absorptiometry, *ECG* electrocardiogram, *eGFR* estimated glomerular filtration rate, *FOBT* faecal occult blood testing, *gGT *gamma-glutamyl transferase, *GHD* growth hormone deficiency, *Gy* gray, *HBV* hepatitis B virus, *HCP* health care provider, *HCV* hepatitis C virus, *HNPCC* hereditary non-polyposis colorectal cancer, *HP* hypothalamic-pituitary, *HSCT* haematopoietic stem cell transplantation, *Hz *Hertz, *IGHG *International Late Effects of Childhood Cancer Guideline Harmonization Group, *IT* intrathecal, *IV* intravenous, *LH/FSHD* luteinising hormone/follicle-stimulating hormone deficiency, *LTFU* long-term follow-up, *MIBG* iodine meta-iodobenzylguanidine, *MRI* magnetic resonance imaging, *NSAIDs* non-steroidal anti-inflammatory drugs, *PedsQL *Pediatric Quality of Life Inventory, *PROMIS* Patient-Reported Outcomes Measurement Information System, *RBC* red blood cell, *SDS* standard deviation score, *TBI* total body irradiation, *TSH* thyroid stimulating hormone, *TSHD* thyroid-stimulating hormone deficiency, *ULN* upper limit of normal

^*^This table includes only the surveillance tests. The full list and details of physical examinations and other surveillance aspects are given in Online Resource 2

^a^Use the following formulas to convert to doxorubicin isotoxic equivalents prior to calculating total cumulative anthracycline dose. Doxorubicin, multiply total dose × 1; daunorubicin, multiply total dose × 0.6 [42]; epirubicin, multiply total dose × 0.8 [42]; idarubicin, multiply total dose × 5 (COG guideline); mitoxantrone, multiply total dose × 10 [43]

^b^If amenorrhoea, measure FSH and oestradiol randomly; if oligomenorrhoea, measure during early follicular phase (days 2–5)

^c^This assessment should be performed after ending oral contraceptive pill/sex steroid replacement therapy use, if applicable, ideally after 2 months of discontinuation

^d^Risk of hypothyroidism for all mentioned exposures. Risk of hyperthyroidism after radiotherapy to a volume exposing the thyroid gland, including TBI, or allogeneic HSCT

^e^MIBG used for diagnostic purposes (e.g. MIBG scanning) does not put patients at risk for hypothyroidism if adequate preventive measures were used

The LTFU care guidelines for the surveillance of hypothalamic-pituitary dysfunction, precocious puberty and ear problems included recommendations for the period after the end of cancer treatment and were therefore adopted. The LTFU recommendations for cardiomyopathy, pericardial and valvular heart disease, subsequent CNS neoplasms, male fertility problems, overweight and obesity, premature ovarian insufficiency and reduced bone mineral density were adopted without change.

### Differences between recommendations for LTFU care and short-term surveillance for health problems

Several recommendations have been adjusted based on evidence and clinical knowledge regarding the timing and/or frequency of surveillance. Where the international guidelines recommend starting surveillance at entry into LTFU care, this has been extrapolated to start at the end of treatment. For pulmonary problems, the LTFU guideline suggests a pulmonary function test at the start of LTFU care. The short-term surveillance guideline suggests that a pulmonary function test should be performed either at the end of treatment or at the age of 6 years, whichever occurs last, depending on the clinical possibility of performing the tests.

In terms of surveillance frequency, the recommendations for physical examination and symptom history for craniofacial growth problems and osteonecrosis have been changed from at least every 5 years to at least annually. These adjustments reflect the urgency of these problems and recognise the likely impact of the pubertal phase, particularly for survivors within the < 5-year timeframe. In addition, osteonecrosis is more likely to occur in the first few years after relevant treatment (particularly with steroids). Similarly, the frequency of surveillance for thyroid function problems to conduct follow-up tests after radioiodine/MIBG therapy has been increased to assessments at 1, 3, 6 and 12 months after radioiodine/MIBG therapy and then annually for survivors ≤ 18 years of age, and at least every 2 years for survivors > 18 years of age.

In addition, for asymptomatic coronary artery disease, subsequent colorectal cancer, dyslipidaemia, thyroid cancer and subsequent female breast cancer, the recommended age to start surveillance is not relevant for the period between the end of treatment and 5 years after diagnosis. Therefore, these health problems do not require surveillance testing during the short-term follow-up period. However, although the incidence of subsequent neoplasms and coronary artery disease is relatively low in the first few years after diagnosis, it is still important for all CAYA cancer survivors to be aware of their potential increased risk and to report any new symptoms promptly.

## Discussion

**Fig. 1:**
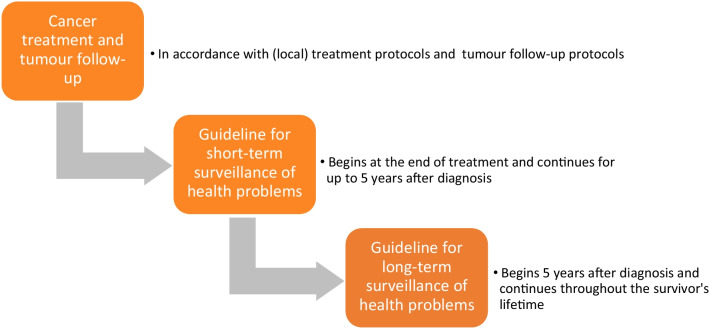
Visualisation of the cancer care continuum: from cancer treatment to follow-up care

Providing a continuum of survivorship care for CAYA cancer survivors, starting at the end of treatment, is critical to improving their quality of life and overall health (see Fig. 1).

To date, current guidelines for survivorship care only cover survivors more than 5 years after diagnosis. This paper describes the work of the PanCareSurPass project to develop a guideline for short-term surveillance of health problems, focusing on addressing the knowledge gap in appropriate guidance for survivors immediately after treatment up to 5 years post-diagnosis. This guideline differs from existing tumour follow-up protocols, which monitor cancer recurrence, by focusing solely on the early identification of health problems caused by cancer treatment so that they can be treated appropriately. Moreover, many cancer treatment protocols assess potential treatment complications as a secondary objective. The guideline for short-term surveillance of health problems is not intended to replace these objectives but provides a minimum set of recommendations. Protocol groups decide their own approach, but we suggest that they start with these recommendations.

To develop the guideline for short-term surveillance of health problems, a working group of survivorship care experts and CAYA cancer survivor representatives collaborated using existing evidence-based IGHG and consensus-based PanCare recommendations for LTFU care. The short-term surveillance guideline covers both physical and mental health problems, as well as preventive measures such as health promotion and education. Ultimately, these recommendations are intended to be incorporated into the follow-up care of all CAYA cancer survivors and can be implemented in follow-up care plans such as the SurPass. Incorporating these recommendations into short-term follow-up and LTFU care plans aims to provide survivors, their families and healthcare professionals, particularly general practitioners, with the knowledge and tools necessary to make personalised recommendations through shared decision-making. This approach empowers survivors and their families to take responsibility for their health care, increases their understanding of health issues, and improves their quality of life [40, 41].

Currently, the IGHG guidelines focus predominantly on LTFU care, with the exception of ototoxicity [25], precocious puberty and hypothalamic-pituitary axis dysfunction [37]. For future updates of surveillance guidelines, it will be essential that guideline panels focus their literature searches and recommendations for surveillance of late health problems on both short-term and long-term follow-up. Furthermore, it is important to note that our expert panel represented the European perspective. Adaptations may be needed to ensure that this guideline is applied in other parts of the world.

In summary, the PanCare guideline for short-term surveillance for health problems, which covers the period from the end of treatment to 5 years after diagnosis, contains 44 recommendations covering awareness, history, physical examination and surveillance testing. The PanCare recommendations for short-term surveillance for health problems and the LTFU care guideline will serve as valuable tools for healthcare professionals to provide post-treatment follow-up care for CAYA cancer survivors. The implementation of these recommendations into individualised care plans and eHealth solutions such as SurPass is essential to provide high-quality, person-centred follow-up care and will improve the quality of life and care for CAYA cancer survivors worldwide.

## Supplementary information

Below is the link to the electronic supplementary material.

Supplementary file1 (PDF 583 KB)
