# Supplementary material for: European recommendations for short-term surveillance of health problems in childhood, adolescent and young adult cancer survivors from the end of treatment to 5 years after diagnosis: a PanCare guideline
Source: J Cancer Surviv. 2023 Dec 4;19(2):603–13. doi: 10.1007/s11764-023-01493-z (PMC11925970; doi:10.1007/s11764-023-01493-z)
Supplement: Supplementary file 1 — Supplementary file1 (PDF 583 KB) [file 11764_2023_1493_MOESM1_ESM.pdf]

European recommendations for short-term surveillance of health problems in childhood, adolescent and young adult cancer survivors from the end of treatment to five years after diagnosis: a PanCare guideline [Journal of Cancer Survivorship]

Ismay A. E. de Beijer[a], Roderick Skinner[b], Riccardo Haupt[c], Desiree Grabow[d], Edit Bardi[e], Andrea Beccaria[c], Adela Cañete Nieto[f], Samira Essiaf[g], Anna-Liesa Filbert[d], Hannah Gsell[h], Anita Kienesberger[h], Thorsten Langer[i], Patricia McColgan[j], Monica Muraca[c], Jelena Rascon[k], Ramona Tallone[c], Zuzana Tomasikova[h], Anne Uyttebroeck[l], Leontien C.M. Kremer[a, m, n], Helena J. H. van der Pal[a], and Renée L. Mulder[a]*, on behalf of the PanCareSurPass consortium[#]*

*Shared last authors

[a] Princess Maxima Center for Pediatric Oncology, Utrecht, The Netherlands, [b] 1) Newcastle University Centre for Cancer, Wolfson Childhood Cancer Research Centre, Herschel Building, Brewery Lane, Newcastle Upon Tyne, NE1 7RU, United Kingdom, 2) Great North Children's Hospital, Royal Victoria Infirmary, Queen Victoria Road, Newcastle Upon Tyne, NE1 4 LP, United Kingdom, 3) Translational and Clinical Research Institute, Wolfson Childhood Cancer Research Centre, Herschel Building, Brewery Lane, Newcastle Upon Tyne, NE1 7RU, United Kingdom, [c] IRCSS Istituto Giannina Gaslini, Genova, Italy, [d] Division of Childhood Cancer Epidemiology / German Childhood Cancer Registry, Institute of Medical Biostatistics, Epidemiology and Informatics (IMBEI), University Medical Centre of the Johannes Gutenberg University Mainz, Mainz, Germany, [e] St Anna Children's Hospital, Vienna, Austria and Department of Paediatrics and Adolescent Medicine, Johannes Kepler University Linz, Austria, [f] Hospital Universitario y Politécnico La Fe, Valencia, Spain, [g] European Society for Paediatric Oncology, C/o BLSI, Clos Chapelle-aux-Champs 30, Bte 1.30.30, Brussels, Belgium, [h] CCI Europe, Vienna, Austria, [l] Universitatsklinikum Schleswig-Holstein, campus Lubeck, Germany, [j] Childhood Cancer Ireland, Carmichael House, 4 Brunswick Street NorthDublin, D07 RHA8, Ireland, [k] Vilnius University Hospital Santaros Klinikos, Vilnius, Lithuania, [l] University Hospitals Leuven, KULeuven, Belgium, [m] University Medical Center Utrecht, Wilhelmina Children's Hospital, Utrecht, the Netherlands, [n] Emma Children's Hospital, Amsterdam UMC, University of Amsterdam, Department of Pediatrics, Amsterdam, The Netherlands

Correspondence to:

Ismay A.E. de Beijer, i.a.e.debeijer-3@prinsesmaximacentrum.nl, Princess Máxima Centre for Pediatric Oncology, Heidelberglaan 25, 3584 CS, Utrecht, the Netherlands

Supplementary Table 1: Overview and comparison recommendations for short-term surveillance for health problems as compared to LTFU care recommendations.

**Short-term recommendation for surveillance of:**	**Who is at risk?**<br>**CAYA cancer survivors treated with or with a history of …**	**What surveillance test should be used, when should it be initiated and at what frequency?** [a]	**What should be done if abnormalities are identified?**	**Changes short-term surveillance recommendations versus LTFU care recommendations**
Awareness only				
Alopecia<br>*Consensus-based PanCare recommendations*	• Chemotherapy<br>• Radiotherapy<br>• HSCT	• Awareness	• Discuss availability of cosmetic solutions and/or psychological support	No changes
Breast cancer (female)<br>*Updated evidence-based IGHG recommendations*	• Radiotherapy ≥ 10 Gy to a volume exposing the breasts<br>• Upper abdominal field radiation that can extend above the diaphragm likely exposing breast tissue at a young age [b]	• Awareness	• Refer to the appropriate HCP	Specific age threshold for surveillance with test (≥ 25 years of age or ≥ 8 years from radiation, whichever occurs last) not relevant for the period of end of treatment until 5 years after diagnosis.
Cerebrovascular problems<br>*Consensus-based PanCare recommendations*	· Radiotherapy to a volume exposing the head, brain or neck, including TBI	· Awareness	· Discuss the importance of controlling cardiovascular and stroke risk factors (hypertension, diabetes, dyslipidaemia, obesity, smoking, alcohol intake and low levels of physical activity)<br>· Perform imaging as appropriate and/or refer to a neurologist, neurosurgeon or vascular specialist	No changes

Colorectal cancer<br>*Consensus-based PanCare recommendations*	· Radiotherapy to a volume exposing the colon and rectum, including TBI	· Awareness	· Refer to the appropriate HCP	Specific age threshold for surveillance with test (5 years after radiation or at the age of 30 years, whichever occurs last) not relevant for the period of end of treatment until 5 years after diagnosis.
Coronary artery disease (asymptomatic)<br>*Evidence-based IGHG recommendations*	· Radiotherapy to a volume exposing the heart	· Awareness	· Timely management of all modifiable cardiovascular disease risk factors (hypertension, diabetes,<br>· dyslipidaemia, overweight or obesity, smoking, alcohol intake and low levels of physical activity) according to local or national guidelines	Specific age threshold for surveillance with test (40 years) not relevant for the period of end of treatment until 5 years after diagnosis.
Dental and oral problems<br>*Consensus-based PanCare recommendations*<br><br>(including dental caries, dental developmental problems (especially if treated at a young age or having suffered from poor nutritional condition), xerostomia and periodontal disease)	· Radiotherapy to a volume exposing the oral cavity or salivary glands, including TBI<br>· Allogeneic HSCT<br>· Chemotherapy	· Awareness	· Refer to specialist dental care or orthodontist if there are significant dental problems related to previous treatment	No changes
Dyslipidaemia<br>*Consensus-based PanCare recommendations*	· TBI<br>· HSCT	· Awareness	· Evaluate other features of metabolic syndrome [c]<br>· Refer to appropriate HCP	Specific age threshold for surveillance with test (40 years) not relevant for the period of end of treatment until 5 years after diagnosis.[d]

Gastro-intestinal problems<br>*Consensus-based PanCare recommendations*	• Radiotherapy to a volume exposing the gastro-intestinal tract, including TBI<br>• Oesophageal or abdominal surgery<br>• cGvHD	• Awareness	• Perform appropriate diagnostic tests and/or refer to a surgeon or gastro-enterologist	No changes
Higher risk groups<br>*Consensus-based PanCare recommendations*	• CNS tumours<br>• Hereditary cancer syndrome<br>• Significant acute toxicity during treatment<br>• Allogeneic HSCT	• Awareness	• A comprehensive approach should be used to identify and address any relevant problems<br>• Survivors with (a suspicion of) a hereditary cancer syndrome should receive additional consultation by a clinical geneticist	No changes
Peripheral neuropathy<br>*Consensus-based PanCare recommendations*	• Vinca-alkaloids<br>• Cisplatin or carboplatin	• Awareness	• Refer to the appropriate HCP<br>• Consider medication for painful neuropathy	No changes
Thyroid cancer<br>*Evidence-based IGHG recommendations*	• Radiotherapy to a volume exposing the thyroid gland, including TBI<br>• MIBG therapy (I-131 MIBG therapy)	• Awareness	• Refer to the appropriate HCP	Specific age threshold for surveillance with test (> 5 years after end of treatment) not relevant for the period of end of treatment until 5 years after diagnosis.
Awareness, history and/or physical examination without surveillance test				

Craniofacial growth problems<br>*Consensus-based PanCare recommendations*	· Radiotherapy to a volume exposing the craniofacial area, including TBI, especially after higher doses and at a young age<br>· Surgery to the face, especially at a young age	· A physical examination for craniofacial growth problems at least yearly starting at end of treatment	· Refer to a reconstructive craniofacial surgeon if craniofacial growth problems are identified<br>· Perform a psychosocial history with specific attention to adjustment difficulties and refer to a psychologist if clinically indicated	Acute problem so more frequent surveillance (yearly) is needed, based on clinical expertise from the guideline panel
Eye problems<br>*Consensus-based PanCare recommendations*<br><br>(including cataract, lacrimal duct atrophy, xerophthalmia, keratitis, telangiectasias, retinopathy, optic chiasm neuropathy, chronic painful eye, maculopathy, papillopathy, visual field deficits and glaucoma)	· Radiotherapy to a volume exposing the eye and orbit, including TBI<br>· Radioiodine therapy (I-131 ablation therapy) (at risk for lacrimal duct atrophy)<br>· Prolonged corticosteroids as anti-cancer treatment, at least 4 weeks continuously (only for cataract)	· A history with specific attention to symptoms of cataract and/or other problems of the eye and orbit at least every 5 years starting at end of treatment<br>· A physical eye examination for external eye abnormalities at least every 5 years starting at end of treatment	· Refer to an ophthalmologist or ocular specialist	No changes

Fatigue (cancer-related) [e]<br>*Evidence-based IGHG recommendations*	· All CAYA cancer survivors are at risk for cancer-related fatigue<br><br>Note: main risk factors are psychological distress, late effects or health problems, pain, older age at follow-up and radiotherapy.	· Medical history focused on survivors' feelings of tiredness and exhaustion [f] regularly (at every follow-up visit, or at general medical check-ups)	*If indication for cancer-related fatigue from history:*<br>· Perform further testing with a validated fatigue measure (e.g. PROMIS Pediatric Fatigue Measure or the PedsQL Multidimensional Fatigue Scale)<br>· Screen for physical problems that may cause fatigue [g]<br><br>*If abnormalities are identified:*<br>· Refer to a specialist in fatigue (or more generic specialist such as a psychologist, physiotherapist, or other relevant specialist)<br>· Discuss useful interventions [h]	No changes
Health promotion<br>*Consensus-based PanCare recommendations*	· All CAYA cancer survivors might benefit from health promotion	· Height, weight and BMI measurement every year in survivors ≤ 18 years of age, and at least every 5 years in survivors > 18 years of age<br>· Blood pressure measurement at least every 5 years starting at end of treatment<br>· Ensure that appropriate immunisations have been given on recovery from active anti-cancer treatment, and that necessary booster immunisations [i] are planned, according to local and national policies<br>at least every 5 years	To promote health, consider referral:<br>· To a physical or occupational therapist if the survivor has special needs and might need to adapt the physical activities for success<br>· To a dietician or refer for a combined lifestyle intervention for weight management<br>· To the appropriate HCP depending on the possible cause of the hypertension<br>· For healthy lifestyle interventions if the survivor wants to participate and if such interventions are available	No changes

		· Give advice, including:<br>- Maintain a physically active lifestyle<br>- Maintain a healthy weight<br>- Eat a healthy diet, according to the current national guidelines<br>- Use adequate sun protection measures<br>- Attend regular six-monthly or yearly dental examinations<br>- Quit smoking and/or reduce exposure to second-hand smoke<br>- Avoid alcohol excess		
Lower urinary tract problems<br>*Consensus-based PanCare recommendations*	· Cyclophosphamide<br>· Ifosfamide<br>· Radiotherapy to a volume exposing the bladder, including TBI<br>· Cystectomy (follow-up by an appropriate specialist)<br>· Hysterectomy<br>· Pelvic surgery<br>· Spinal cord surgery	· A history with specific attention to urinary tract symptoms at least every 5 years starting at end of treatment	· Perform a urinalysis including cytology and urine culture<br>· Refer to a urologist if the urinalysis results are abnormal	No changes

Melanoma and non-melanoma skin cancer<br>*Consensus-based PanCare recommendations*	· Any radiotherapy, including TBI, predominantly in the radiotherapy field<br>· Allogeneic HSCT, especially with a history of skin GvHD	· Self-examination for new spots and changing moles, at least every 6 months<br>· History at least every 2 years<br>· Skin examination at least every 2 years starting at end of treatment	· Refer to a dermatologist	No changes
Mental health problems<br>*Evidence-based IGHG guideline*<br><br>(including anxiety, behavioural problems, depression, post-traumatic stress and suicidal ideation)	· All CAYA cancer survivors are at risk for mental health problems	· A history with focus on survivors' mental health [j] at every long-term follow-up visit or general medical check-up [k]	*Survivors who indicate mental health symptoms:*<br>· Prompt referral to psychiatrist, psychologist, or social worker for further diagnostic and risk assessment<br><br>*Survivors who indicate suicidal ideation:*<br>· Immediate referral to psychiatrist, psychologist, or local mental health crisis services	No changes

Neurocognitive problems<br>*Consensus-based PanCare recommendations*<br><br>(including problems in the cognitive domains of academic and school performance, attention, executive functions, intelligence, language, memory, processing speed or visual-motor integration)	· A CNS tumour<br>· Brain surgery<br>· Radiotherapy to a volume exposing the brain, including TBI<br>· High dose cytarabine IV<br>· High dose methotrexate IV<br>· Any chemotherapy IT especially if the survivor was treated at a young age	· A history with specific attention to educational and/or vocational progress or decline<br>- at least every 2 years in survivors ≤ 18 years of age<br>- at least every 5 years in survivors > 18 years of age<br>starting at end of treatment	· Refer to a (neuro)psychologist for a formal neuropsychological evaluation	No changes
Obstetric problems (female)<br>*Evidence-based IGHG recommendations*	· Radiotherapy to a volume exposing the uterus	*All female survivors at risk of reproductive age:*<br>· Discuss the risk of adverse obstetric outcomes (miscarriage, premature birth, low birth weight; but not congenital anomalies)<br>· High-risk obstetric surveillance during pregnancy	· Refer to the appropriate HCP	No changes
Osteonecrosis<br>*Consensus-based PanCare recommendations*	· Prolonged corticosteroids as anti-cancer treatment, at least 4 weeks continuously<br>· HSCT, especially with any history of cGvHD	· A history for symptoms of osteonecrosis at least yearly, starting at end of treatment	· Suspicion of osteonecrosis should always be followed by a timely referral to an orthopaedic surgeon	Acute problem so more frequent surveillance (yearly) is needed, based on clinical expertise from the guideline panel

	• High dose radiotherapy involving any part of the skeleton			
Pain (chronic)<br>*Consensus-based PanCare recommendations*	• All CAYA cancer survivors are at risk for chronic pain	• A screening question for pain, asking for the presence of (chronic) pain, at least every 5 years starting at end of treatment	• Perform a more extensive pain history (including location, intensity, relation to cancer treatment, changes over time and interference with daily or social activities), for example using the Brief Pain Inventory<br>• Refer to the appropriate HCP	No changes
Psychosocial problems<br>*Evidence-based IGHG-recommendations*<br><br>(including dependent living, educational problems, relationship problems, social withdrawal, under-employment or unemployment)	• All CAYA cancer survivors are at risk for psychosocial problems	• A history focused on educational progress [l] and/or vocational planning and employment status [m] and social withdrawal, at every long-term follow-up visit or general medical check-up [k], at least annually until education is completed	• Educational and/or vocational problems should be documented in the survivor's medical records and shared with all members of the survivor's team<br>• Refer to a psychologist for further diagnostic testing and treatment<br>• Refer to appropriate social worker or educational professional or vocational counselor as required	No changes

Spine scoliosis and kyphosis<br>*Consensus-based PanCare recommendations*	· Surgery of the spine<br>· Surgery of the chest (not including central venous catheter placement)<br>· Radiotherapy to a volume exposing the spine<br>· Spinal or paraspinal malignancies<br>· Malignancies of bones of the lower limbs	· A physical examination of the spine every year until growth is completed, starting at end of treatment; the surveillance frequency may be increased during puberty	· Perform imaging and/or refer to an orthopaedic surgeon or physical therapist as clinically indicated	No changes
Spleen problems (overwhelming bacterial infections)<br>*Consensus-based PanCare recommendations*	· Splenectomy<br>· Radiotherapy ≥ 10 Gy to a volume exposing the spleen<br>· Allogeneic HSCT (conditioned with or without TBI)<br>· Autologous HSCT (conditioned with TBI)	· Educate about events that necessitate immediate start of therapeutic antibiotics and prompt evaluation by a HCP [v]<br>· Ensure that therapeutic antibiotics are readily available<br>· Advise wearing medical bracelet or carrying patient card<br>· Discuss importance of seeking expert advice when travelling to endemic areas	*In case of fever > 38.3 °C, infective or septic symptoms, or animal or human bite with skin break:*<br>· Arrange prompt evaluation by a HCP including a physical examination, blood count and blood culture<br>· Immediately treat with therapeutic antibiotics according to local and national policies until blood culture results are available	No changes
Subsequent neoplasms<br>*Consensus-based PanCare recommendations*<br><br>(including AML, bladder cancer, bone cancer, breast cancer, CNS	· All CAYA cancer survivors are at a potential increased risk depending on the treatment that they have received and their genetic risk profile.	*General advice:*<br>· Discuss the importance of prompt reporting of new symptoms or masses<br>· Discuss healthy lifestyle recommendations<br>· Encourage reduction of risk behaviour (smoking, alcohol	· Perform the appropriate diagnostic tests<br>· Refer to the appropriate HCP	Note I added based on strong clinical expertise from the guideline panel

neoplasms, colorectal cancer, lung cancer, oral cancer, melanoma and non-melanoma skin cancer and thyroid cancer [n])	Note I: Survivors must be aware that subsequent neoplasms are relatively unlikely to occur within the first five years after end of treatment.<br><br>Note II: Consult the IGHG guidelines for breast cancer, CNS neoplasms and thyroid cancer, and newly developed recommendations for colorectal cancer and melanoma and non-melanoma skin cancer to determine whether the survivor is at increased risk for these malignancies and would benefit from specific surveillance strategies.	consumption, drug use, sun exposure)<br>· Encourage HPV vaccination (according to national guidelines) and consider advising safe sexual practices<br>· Encourage participation in the national cancer screening programmes, unless more intensive or earlier surveillance is specified in the guidelines [n]<br><br>*Surveillance strategy in all survivors:*<br>· Family history of malignancies, at least every 5 years<br><br>*Surveillance strategy in survivors with, or with a suspicion of, a hereditary cancer syndrome [o]:*<br>· Additional consultation by a clinical geneticist to determine individualised surveillance methods and frequency at entry into follow-up		
Awareness, history and/or physical examination with potential surveillance test				

CNS neoplasms<br>*Evidence-based IGHG recommendations*<br><br>(including meningiomas, (high-grade) gliomas and other CNS neoplasms)	· Radiotherapy to a volume exposing the head or brain, including TBI	· Inform about symptoms and signs [p] that may be related to a subsequent CNS neoplasm<br>· Neurologic history at every follow-up visit, which may be at 1-5 year intervals<br>· Neurologic examination at every follow-up visit, which may be at 1-5 year intervals<br><br>Note: No recommendation can be formulated for routine MRI surveillance for asymptomatic survivors. The decision to undertake MRI surveillance should be made by the CAYA cancer survivor and HCP after careful consideration of the potential harms and benefits of MRI surveillance.	· Refer to the appropriate HCP	No changes
Awareness, history and/or physical examination with surveillance test				

Cardiac problems (arrhythmia)<br>*Consensus-based PanCare recommendations*	· Radiotherapy ≥ 15 Gy to a volume exposing the heart<br>· Anthracyclines, including doxorubicin, daunorubicin, epirubicin, idarubicin and mitoxantrone	· A cardiac history at least every 5 years, starting at end of treatment<br>· A physical cardiac exam at least every 5 years, starting at end of treatment<br>· ECG once at the end of treatment<br>· Repeat ECG once after the age of 18 years if end of treatment was at a younger age	· Refer to a cardiologist	Suggested extrapolation to start of surveillance based on clinical expertise from the guideline panel
Cardiac problems (cardiomyopathy)<br>*Evidence-based IGHG recommendations*	· Radiotherapy ≥ 15 Gy to a volume exposing the heart<br>· Anthracyclines, including doxorubicin, daunorubicin, epirubicin, idarubicin and mitoxantrone	· A cardiac history at least every 5 years, starting at end of treatment<br>· A physical cardiac exam at least every 5 years, starting at end of treatment<br>· Echocardiogram with assessment of left ventricular systolic function:<br>- Radiotherapy ≥ 30 Gy to a volume exposing the heart: twice every 5 years, starting no later than 2 years after cardiotoxic therapy<br>- Total cumulative anthracycline dose ≥100 - 250 mg/m^2 [q]: every 5 years, starting no later than 2 years after cardiotoxic therapy<br>- Total cumulative anthracycline dose ≥250 mg/m^2 [q]: twice every 5	· Refer to a cardiologist<br>· Refer for interventions to help avert the risk of symptomatic cardiomyopathy if modifiable cardiovascular risk factors are identified	No changes

		years, starting no later than 2 years after cardiotoxic therapy<br>- Combination of radiotherapy ≥ 15 Gy to a volume exposing the heart and total cumulative anthracycline dose ≥100 mg/m^2 [q]: twice every 5 years, starting no later than 2 years after cardiotoxic therapy<br>- Anthracyclines, mitoxantrone and/or radiotherapy to a volume exposing the heart: prior to pregnancy or in the first trimester. Continuing cardiomyopathy surveillance is reasonable during pregnancy for female survivors treated with anthracyclines or chest RT who had a history of prior LV systolic dysfunction that has resolved even in the presence of a normal baseline ejection fraction in the first trimester.<br>- Screening for modifiable cardiovascular risk factors (hypertension, diabetes, dyslipidaemia, obesity, smoking, alcohol intake, and low levels of physical activity)		

Cardiac problems (pericardial and valvular heart disease)<br>*Consensus-based PanCare recommendations*	· Radiotherapy ≥ 15 Gy to a volume exposing the heart	· A cardiac history at least every 5 years, starting at end of treatment<br>· A physical cardiac exam at least every 5 years, starting at end of treatment<br>· Echocardiogram with specific attention to the pericardium and valvular structure and function: at least every 5 years, starting no later than 2 years after cardiotoxic therapy	· Refer to a cardiologist	No changes
Ear problems<br>*Evidence-based IGHG recommendations*<br><br>(including hearing loss and tinnitus)	· Cisplatin (with or without carboplatin > 1500 mg/m^2)<br>· Radiotherapy ≥ 30 Gy to a volume exposing the head or brain	*Survivors < 6 years of age at risk:*<br>· Extensive testing by audiologist every year, to begin no later than the end of treatment<br><br>*Survivors ≥ 6 years of age at risk*<br>· Pure tone conventional audiometry testing at 1000-8000 Hz<br>· Additional testing with high frequency audiometry > 8000 Hz (whenever equipment is available), to begin no later than the end of treatment<br>- every other year if 6-12 years of age<br>- every 5 years for adolescents and young adults ≥ 12 years of age	· Refer to an audiologist or auditory clinic if there are symptoms suggesting hearing loss, abnormal audiological test results showing a loss of more than 15 dB absolute threshold level (1000-8000 Hz) or if there are symptoms of tinnitus	No changes

Fertility problems and sexual dysfunction (male)<br>*Evidence-based IGHG recommendations*<br><br>(including impaired fertility, impaired spermatogenesis, testosterone deficiency and physical sexual dysfunction)	· Alkylating agents<br>· Radiotherapy to a volume exposing the testes, including TBI<br>· Surgery to the spinal cord, sympathetic nerves or pelvis<br>· Hypogonadism	*All survivors at risk:*<br>· Counselling regarding the risk of impaired spermatogenesis, testosterone deficiency and physical sexual dysfunction (including erectile and ejaculatory dysfunction), and its implications for future health and fertility at the request of the survivor after informed discussion or when paternity is desired in the foreseeable future, at least every 5 years starting at end of treatment<br><br>*Pre- and peri-pubertal survivors treated with radiotherapy ≥ 12 Gy to a volume exposing the testes, including TBI:*<br>· Growth (height) and pubertal development and progression (Tanner stage) at least every year, with increasing frequency as clinically indicated depending on growth and pubertal progress<br><br>Note: Regular growth and pubertal monitoring should be started by no later than 12 years (and no earlier than 10 years) of age.	· Refer to male reproductive medicine, andrology, endocrinology or urology if seeking paternity, unsuccessful attempts to conceive ≥ 6 months (regardless of sperm count) or symptoms suggesting physical sexual dysfunction<br>· Refer to male reproductive medicine, andrology, endocrinology or urology if severe oligospermia (sperm counts ≤ $5x10^6$/ml) or if laboratory results suggest testosterone deficiency	No changes

		Post-pubertal survivors treated with surgery to the spinal cord, sympathetic nerves or pelvis; or radiotherapy to a volume exposing the testes or pelvis; or with hypogonadism:<br>· Sexual history every 5 years<br><br>*Post-pubertal survivors treated with radiotherapy ≥ 12 Gy to a volume exposing the testes, including TBI:*<br>· Early morning testosterone at clinically appropriate time intervals<br>· LH in addition to (early morning) testosterone if clinical signs of hypogonadism, previous low or borderline testosterone concentrations, or if an early morning testosterone sample cannot be obtained, at least every 2-3 years<br><br>*Post-pubertal survivors at risk that desire assessment of potential for future fertility:*<br>· Semen analysis		

Hypertension<br>*Consensus-based PanCare recommendations*	· Radiotherapy to a volume exposing the kidneys, or to a volume exposing the heart and associated large vessels, including TBI<br>· Nephrectomy<br>· Ifosfamide<br>· Platinum based chemotherapy<br>· Nitrosoureas<br>· Immunosuppressives, e.g., ciclosporin, tacrolimus	· Blood pressure measurement at least every 2 years and at every follow-up visit	· Evaluate other features of metabolic syndrome [c]<br>· Refer to appropriate HCP depending on the possible cause of the hypertension	No changes
Hypothalamic-pituitary axis problems<br>*Evidence-based IGHG recommendations*<br><br>(including GHD, TSHD, LH/FSHD and ACTHD)	· Radiotherapy to a volume exposing the HP region, including TBI (if ≥ 30 Gy, refer directly to (paediatric) endocrinologist or see in multidisciplinary team)<br>· Surgery near or within the HP region (refer directly to (paediatric) endocrinologist or see in multidisciplinary team)<br>· A CNS tumour near or within the HP region (refer directly to (paediatric) endocrinologist or see in multidisciplinary team)	*Pre-pubertal and peri-pubertal survivors at risk:*<br>· Relevant clinical history for HP axis problems<br>· Physical examination for symptoms and signs suggestive of HP axis problems<br>· Height velocity in relation to parental height<br>· Tanner stage (note: boys exposed to gonadotoxic therapy (e.g. alkylating agents and radiotherapy to the testes) may have testes small for pubertal stage while in puberty)<br>every 6 months, at ≥ 1 year after completion of radiotherapy or directly	· Refer to (paediatric) endocrinologist if:<br>- a pre- or peri-pubertal survivor experiences decline in height velocity, or lack of acceleration of growth velocity in case of signs of puberty, or a height SDS below their target height range SDS, which cannot be explained by other causes<br>- there are clinical symptoms or laboratory results suggestive for HP axis problems<br>- a low morning cortisol is found [r]<br>· Counsel survivors with a low morning cortisol regarding the risks associated with untreated ACTHD. Hydrocortisone replacement therapy should be	No changes

	· Hydrocephalus or CSF shunt (at risk for GHD)	after hydrocephalus or CSF shunt occurrence<br><br>· fT4, TSH, morning cortisol every year, starting , at ≥ 1 year after completion of radiotherapy or directly after hydrocephalus or CSF shunt occurrence<br><br>*Post-pubertal survivors at risk:*<br>· Relevant clinical history for HP axis problems<br>· Physical examination for symptoms and signs suggestive of HP axis problems<br>· Evaluation of menstrual cycle (females)<br>· fT4, TSH, morning cortisol, IGF-1<br>· Morning testosterone, or free testosterone if overweight, and LH (males)<br>· Estradiol, FSH and LH (females)<br>every year, starting at ≥ 1 year after radiotherapy or directly after hydrocephalus or CSF shunt occurrence<br><br>Note: an IGF-1 level even as high as 0 SDS does not rule out GHD.	prescribed during periods of physiologic stress until provocative testing has established adequate ACTH axis function upon referral to endocrine services.<br>· Counsel survivors with (a suspicion of) HP axis problems regarding the benefits of hormonal replacement therapy on overall health, as well as the risks associated with untreated HP axis problems, and assist them with coordinating and obtaining an early referral when appropriate. Note that thyroid hormone treatment should be started only after evaluation and approval of function of the ACTH axis.	

		Note: continue surveillance at least 15 years from exposure. Continuation of surveillance should be a shared decision between survivor and HCP considering available health care resources. If surveillance is terminated, the survivor should be educated about possible signs and symptoms of HP axis problems.		
Impaired glucose metabolism and diabetes mellitus<br>*Consensus-based PanCare recommendations*	· Radiotherapy to a volume exposing the pancreas, including TBI	· Fasting blood glucose with or without HbA1c at least every 5 years starting at end of treatment	· Evaluate other features of metabolic syndrome [c]<br>· Refer to appropriate HCP	Suggested extrapolation to start of surveillance based on clinical expertise from the guideline panel
Iron overload<br>*Evidence-based IGHG recommendations*	· HSCT<br>· Multiple red blood cell transfusions	· Serum ferritin, once at end of treatment	*In case of increased serum ferritin (>500 ng/ml):*<br>· Repeat test within 6 months<br><br>*If persistent abnormal serum ferritin levels (>500 ng/ml):*<br>· Perform a MRI T2* to quantify the liver iron content<br><br>*If confirmed elevated liver iron content:*<br>· Refer to a hematologist or other specialist to start treatment, such as phlebotomy or chelation therapy	Suggested extrapolation to start of surveillance based on clinical expertise from the guideline panel

Late liver injury<br>*Evidence-based IGHG recommendations*<br><br>(including liver fibrosis or cirrhosis, hepatocellular liver injury, hepatobiliary dysfunction, biliary tract injury or liver synthetic dysfunction)	· Radiotherapy to a volume exposing the liver, including TBI<br>· HSCT<br>· Methotrexate<br>· Mercaptopurine<br>· Thioguanine<br>· Dactinomycin<br>· Busulfan<br>· Sinusoidal obstruction syndrome<br>· cGvHD<br>· Liver surgery<br>· Chronic viral hepatitis (follow-up by appropriate specialist, e.g. hepatologist or infectious disease specialist, according to local or national hepatitis CPGs)	· Physical examination for height, weight, BMI and signs of liver disease or bile duct injury (i.e. hepatosplenomegaly, spider naevi or pruritus)<br>· Serum liver enzyme concentrations (ALT, AST, gGT, ALP) once at end of treatment	*In case of increased liver enzyme values:*<br>· Between 1-2 x ULN: repeat the test within 1 year.<br>· > 2x ULN: repeat the test within 2 months.<br><br>*In case of persistent liver abnormalities (> ULN):*<br>· Refer to a hepatologist or gastroenterologist for further examination if there is no obvious explanation (alcohol, medication, obesity)<br>· Avoid or prescribe with caution potentially hepatotoxic medications and supplements<br>· Evaluate body mass index and discuss healthy weight goals, especially in those with evidence of metabolic syndrome<br>· Consider immunization against hepatitis A and B if not already immune<br>· Counsel about importance of measures to maintain liver health:<br>Cautious use or avoidance of alcohol intake<br>Maintain a healthy weight and lifestyle<br>Precautions to reduce viral transmission to household and sexual contacts in survivors with chronic HBV/HCV infection	Suggested extrapolation to start of surveillance based on clinical expertise from the guideline panel

Overweight and obesity<br>*Consensus-based PanCare recommendations*	· Hypothalamic or pituitary tumour<br>· Radiotherapy to a volume exposing the hypothalamus or pituitary gland, including TBI<br>· Neurosurgery of hypothalamus or pituitary gland	· Height, weight and BMI at least every 2 years and at every follow-up visit	· Evaluate other features of metabolic syndrome [c]<br>· Refer to dietician or refer for a combined lifestyle intervention for weight management<br>· Consider referral to appropriate HCP<br>· Consider referral to endocrinologist for evaluation and management of central endocrinopathies	No changes
Precocious puberty (central)<br>*Evidence-based IGHG recommendations*	· Radiotherapy to a volume exposing the HP region, including TBI (if ≥ 30 Gy, refer directly to (paediatric) endocrinologist or see in multidisciplinary team)<br>· Surgery near or within the HP region (refer directly to (paediatric) endocrinologist or see in multidisciplinary team)<br>· A CNS tumour near or within the HP region (refer directly to (paediatric) endocrinologist or see in multidisciplinary team)<br>· Hydrocephalus or cerebrospinal fluid shunt	*All survivors at risk:*<br>· Relevant clinical history for symptoms of central precocious puberty<br>· Physical examination for signs of central precocious puberty<br>· Height velocity in relation to parental height<br>· Tanner stage<br>every 6 months, starting at ≥ 1 year after completion of radiotherapy or directly after hydrocephalus or CSF shunt occurrence<br><br>*Male survivors at risk below 9 years exposed to gonadotoxic treatment:*<br>· Morning testosterone<br>every year, starting at ≥ 1 year after completion of radiotherapy or	· Refer to a paediatric endocrinologist if there are clinical symptoms and signs suggestive for central precocious puberty, or if morning testosterone is abnormal<br>· Counsel survivors with (a suspicion of) central precocious puberty on overall health as well as the risk for short stature associated with untreated central precocious puberty, and assist them with coordinating and obtaining an early referral when appropriate	No changes

		directly after hydrocephalus or CSF shunt occurrence<br><br>Note: Continue surveillance until the age of 8 years for girls and 9 years for boys. Boys exposed to radiotherapy to the testes may have testes small for pubertal stage while in puberty. Instead, morning testosterone (before 10:00 AM) should be used as screening modality as testicular volume may be unreliable.		
Premature ovarian insufficiency (female)<br>*Evidence-based IGHG recommendations*<br><br>(including impaired fertility, amenorrhoea and premature menopause)	· Alkylating agents<br>· Radiotherapy to a volume exposing the ovaries, including TBI	*All survivors at risk:*<br>· Counselling regarding the risk of premature ovarian insufficiency and its implications for future fertility, at least every 5 years or more often at the request of the survivor and/or their family (after informed discussion) or when maternity is desired in the foreseeable future<br>· Not recommended: measurement of AMH as primary surveillance modality<br><br>*Pre- and peri-pubertal survivors at risk:*<br>· Monitoring of growth (height) and pubertal	*Pre- and peri-pubertal survivors at risk:*<br>· Refer to paediatric endocrinology or gynaecology for any survivor who has no signs of puberty by 13 years of age (Tanner stage 2 breast development), primary amenorrhoea by 16 years of age, or failure of pubertal progression for ≥ 12 months.<br><br>*Post-pubertal survivors at risk:*<br>· Refer to gynaecology, reproductive medicine or endocrinology females who present with menstrual cycle dysfunction suggesting premature ovarian insufficiency or who desire assessment about potential for future fertility	No changes

		development and progression (Tanner stage) at least every year, with increasing frequency as clinically indicated based on growth and pubertal progression<br>· FSH and oestradiol [z] in case of failure to initiate or progress through puberty at least for girls ≥ 11 years of age, and for girls with primary amenorrhoea (16 years of age)<br><br>*Post-pubertal survivors at risk:*<br>· History and physical examination with specific attention to premature ovarian insufficiency symptoms (amenorrhoea, irregular cycles) every 5 years starting at end of treatment<br>· FSH and oestradiol [r,s] in case of menstrual cycle dysfunction suggesting premature ovarian insufficiency, or if assessment of potential for future fertility is desired	*Pre- and peri-pubertal survivors:*<br>· Consider sex steroid replacement therapy by referral to paediatric endocrinology or gynaecology<br><br>*Post-pubertal survivors:*<br>· Consider sex steroid replacement therapy by referral to endocrinology or gynaecology	

Pulmonary problems<br>*Consensus-based PanCare recommendations*<br><br>(including pulmonary dysfunction and worsening pulmonary fibrosis after high oxygen exposure in survivors treated with bleomycin who already have evidence of pulmonary fibrosis)	· Carmustine (BCNU)<br>· Lomustine (CCNU)<br>· Busulfan<br>· Bleomycin<br>· Radiotherapy to a volume exposing the lungs, including TBI<br>· Allogeneic HSCT<br>· Thoracic surgery	· History with specific attention to pulmonary dysfunction at least every 5 years starting at end of treatment<br>· Physical pulmonary examination at least every 5 years starting at end of treatment<br>· Pulmonary function tests, including spirometry and diffusing capacity for carbon monoxide (DLCO), once at end of treatment or at the age of 6 years, whichever occurs last<br>· Consider pneumococcal vaccination status according to local or national guidelines<br><br>*Other advice:*<br>· Avoid tobacco, quit smoking and/or reduce exposure to environmental smoke	*If initial pulmonary function test is abnormal:*<br>· Consult with or refer to pulmonologist<br><br>*If any abnormalities are identified during subsequent follow-up visits*<br>· Repeat pulmonary function tests<br>· Consult with or refer to pulmonologist if they are abnormal	Suggested extrapolation to start of surveillance based on clinical expertise from the guideline panel

Reduced bone mineral density<br>*Evidence-based IGHG guideline*	· Cranial or craniospinal radiotherapy<br>· TBI<br>· Corticosteroids as anti-cancer treatment, at least 4 weeks continuously<br><br>Other risk factors are:<br>· Hypogonadism<br>· Growth hormone deficiency<br>· Low BMI or underweight Male sex<br>· White race<br>· Lack of physical activity [t]<br>· Current or prior smoking	*Survivors treated with cranial or craniospinal radiotherapy or TBI*:<br>· A history with specific attention to risk factors (poor vitamin D and/or calcium intake, minimal weight-bearing exercise, comorbidities) and symptoms (back pain, fractures) of reduced bone mineral density at least every 5 years, starting at end of treatment<br>· A DXA scan once, 2-5 years after end of treatment, and thereafter as clinically indicated<br><br>Note: It might be considered to postpone the DXA-scan in pre-pubertal and pubertal survivors.<br><br>*Survivors treated with corticosteroids as anti-cancer treatment:*<br>· No recommendation can be formulated for or against BMD surveillance; the decision to undertake surveillance should be made together by the survivor and healthcare provider, after careful consideration of the potential harms and benefits	*In CAYA cancer survivors with a BMD Z-score ≤-2:*<br>· Referral to (or consultation of) a medical bone health specialist [u] is recommended for further (endocrine) evaluation, interpretation of BMD findings, treatment, and follow-up<br><br>*In CAYA cancer survivors with a BMD Z-score ≤-1 and >-2:*<br>· Evaluate for the presence of endocrine defects and consult a medical bone health specialist [t] for further evaluation and interpretation of BMD findings as clinically indicated<br>· Repeat DXA after 2 years, and thereafter as clinically indicated based on BMD change (i.e. in case of BMD decline more than the DXA machine's least significant change) and ongoing risk assessment<br><br>*Other advice to be given:*<br>· Recommend adequate calcium and vitamin D intake, and adequate physical activity according to guidelines for the general population	No changes

Renal problems<br>*Consensus-based PanCare recommendations*<br><br>(including glomerular and tubular dysfunction)	· Ifosfamide<br>· Cisplatin<br>· Carboplatin<br>· Radiotherapy to a volume exposing the kidney or urinary tract, including TBI Nephrectomy<br>· HSCT	*All survivors at risk:*<br>· Glomerular function testing including blood testing (creatinine), urine testing (creatinine, proteinuria), eGFR calculation, at least every 5 years starting at end of treatment<br><br>*Survivors treated with ifosfamide, cisplatin or carboplatin:*<br>· Additional tubular function testing including blood testing (Na, K, Mg, P, Ca, phosphate, albumin) and urine testing (glucose, phosphate) at least every 5 years starting at end of treatment<br><br>*Other advice:*<br>· Education about caution in the use of NSAIDs<br>· Counselling about single kidney-related health risks	· Electrolyte supplementation as guided by serum biochemistry if an electrolyte imbalance is detected<br>· Refer to nephrologist if proteinuria and/or chronic kidney disease are identified	Suggested extrapolation to start of surveillance based on clinical expertise from the guideline panel

Thyroid function problems<br>*Consensus-based PanCare recommendations*<br><br>(including hypothyroidism and hyperthyroidism [w])	· Radiotherapy to a volume exposing the thyroid gland, including TBI<br>· Radioiodine therapy (I-131 ablation therapy)<br>· MIBG therapy (I-131 MIBG therapy) [x]<br>· Allogeneic HSCT<br>· Total thyroidectomy (follow-up by an endocrinologist starting directly after surgery)	· A history with specific attention to hypothyroidism and/or hyperthyroidism<br>· TSH and fT4 measurement<br>- every year in survivors ≤ 18 years of age<br>- at least every 2-3 years in survivors > 18 years of age<br><br>*Frequency after radioiodine/MIBG therapy*:<br>· 1, 3, 6 and 12 month after radioiodine/MIBG therapy (I-131 ablation/MIBG therapy) and afterwards every year in survivors ≤ 18 years of age, and at least every 2 years in survivors > 18 years of age<br><br>*Female survivors at risk of hypothyroidism:*<br>· Discuss the importance of measuring TSH and fT4 prior to attempting pregnancy and periodically during pregnancy at least every 5 years<br>· Measure TSH and fT4 prior to attempting pregnancy and periodically during pregnancy	· Repeat TSH and fT4 within 3 months if results are (borderline) abnormal<br>· Refer to an endocrinologist if results are repeatedly abnormal	Frequency after radioiodine/ MIBG therapy based on clinical expertise from the guideline panel

Note. Language/format changes are not included in the fifth column. Abbreviations: ACTHD = adrenocorticotropic hormone deficiency, ALP = alkaline phosphatase, ALT = alanine aminotransferase, AML = acute myeloid leukaemia, AST = aspartate aminotransferase, CAYA = childhood, adolescent and young adult, cGvHD = chronic graft-versus-host disease, CPG = clinical practice guideline, CNS = central nervous system, dB = decibel, DXA = dual-energy X-ray absorptiometry, ECG = electrocardiogram, eGFR = estimated glomerular filtration rate, FOBT = faecal occult blood testing, gGT = gamma-glutamyl transferase, GHD = growth hormone deficiency, Gy = gray, HBV = hepatitis B

virus, HCP = health care provider, HCV = hepatitis C virus, HNPCC = Hereditary Non-Polyposis Colorectal Cancer, HP = hypothalamic-pituitary, HSCT = haematopoietic stem cell transplantation, HZ = Hertz, IGHG = International Late Effects of Childhood Cancer Guideline Harmonization Group, IT = intrathecal, IV = intravenous, LH/FSHD = luteinising hormone/follicle stimulating hormone deficiency, LH/FSHD = luteinising hormone/follicle stimulating hormone deficiency, MIBG = iodine meta-iodobenzylguanidine, MRI = magnetic resonance imaging, NSAIDs = non-steroidal anti-inflammatory drugs, PedsQL = Pediatric Quality of Life Inventory, PROMIS = Patient-Reported Outcomes Measurement Information System, RBC = red blood cell, SDS = standard deviation score, TBI = total body irradiation, TSH = thyroid stimulating hormone, TSHD = thyroid stimulating hormone deficiency, ULN = upper limit of normal.

[a] Surveillance should be initiated after end of treatment (unless specified otherwise) and surveillance should be continued life-long (unless specified otherwise).

[b] For survivors treated with upper abdominal field radiation that can extend above the diaphragm likely exposing breast tissue at a young age, the surveillance decision should be an individual one, taking into account additional risk factors (patient age, family history, menopausal status, other previous cancer treatment) and personal values regarding the potential advantages and disadvantages of surveillance.

[c] Important features of metabolic syndrome are dyslipidaemia, impaired glucose metabolism, hypertension and overweight.

[d] Timing of initiation and frequency should be based on the intensity of treatment exposure, family history, presence of co-morbid conditions associated with disease risk or by general risk management guidelines.

[e] Cancer-related fatigue is defined as “a distressing, persistent, subjective sense of physical, emotional and/or cognitive tiredness or exhaustion related to cancer or cancer treatment that is not proportional to recent activity and interferes with usual functioning”.

[f] Questions to ask: “Do you get tired easily?”, or “Are you too tired or exhausted to enjoy the things you like to do?”

[g] For example other late effects like cardiac dysfunction, endocrine dysfunction, pulmonary dysfunction, and renal dysfunction (IGHG guidelines under development); and/or other general causes like anaemia, arthritis, neuromuscular complications, pain, fever and/or infection, and nutritional deficiencies (list not conclusive).

[h] Physical activity, education about cancer-related fatigue, relaxation and mindfulness, cognitive behavioural therapy, adventure-based training.

[i] Booster immunisations after standard chemotherapy, re-immunisation after HSCT

[j] Questions to ask: Have you … “Been feeling sad, angry, or less interested in things than usual?”, “Been feeling worried, tense, stressed, or overwhelmed?”, “Had trouble coping with thoughts, memories, or reminders of the cancer experience?”, “Had thoughts of harming yourself or ending your life?”, “Considered connecting with a healthcare provider to support your mental health?”

[k] If survivors are not scheduled for annual visits, screening can be done via phone or telehealth, or can be delegated to a suitable professional in the school of the survivor.

[l] Questions to ask: “Do you have any problems keeping up at school?”, “Has your performance been affected in any way? In what way?”, “Are there certain areas/subjects you struggle with?”, “Are there areas of your education that cause you stress or anxiety?”.

[m] Questions to ask: “What profession would you like to pursue?”, “Have you had difficulties when applying for a job?”, “Do you have any problems keeping up with your work?”, “Do you have any problems keeping a full time job?”.

[n] IGHG guidelines are currently available for surveillance of breast cancer, CNS neoplasms and thyroid cancer. Specific newly developed recommendations are available for colorectal and melanoma and non-melanoma skin cancer.

[o] For example, but not limited to: Fanconi anaemia, dyskeratosis congenita, Li-Fraumeni syndrome (TP53 mutation), neurofibromatosis type I, hereditary non-polyposis colorectal cancer (HNPCC or Lynch syndrome).

[p] Progressively worsening, severe, unrelenting headaches, new onset cognitive, motor, sensory or behavioral changes, balance problems, seizures, and other neurologic deficits.

[q] Use the following formulas to convert to doxorubicin isotoxic equivalents prior to calculating total cumulative anthracycline dose. Doxorubicin: multiply total dose x 1; Daunorubicin: multiply total dose x 0.6 (Feijen, 2019); Epirubicin: multiply total dose x 0.8 (Feijen, 2019); Idarubicin: multiply total dose x 5 (COG guideline); Mitoxantrone: multiply total dose x 10 (Feijen, 2019).
References: EAM Feijen, WM Leisenring, KL Stratton et al. Derivation of anthracycline and anthraquinone equivalence ratios to doxorubicin for late-onset cardiotoxicity. JAMA Oncology. 2019;5(6):864-871.
EAM Feijen, A Font-Gonzalez, HJH van der Pal et al. Risk and temporal changes of heart failure among 5-year childhood cancer survivors: a DCOG-LATER study. J Am Heart Assoc. 2019; 8(1):e009122.

[r] These survivors should be counselled regarding the risks associated with untreated ACTH deficiency. A hydrocortisone stress scheme should be provided in case of doubt of an adequate functioning ACTH axis.

[r] If amenorrhoea, measure FSH and oestradiol randomly; if oligomenorrhoea, measure during early follicular phase (day 2-5).

[s] This assessment should be performed after ending oral contraceptive pill/sex steroid replacement therapy use, if applicable, ideally after two months discontinuation.

[t] The WHO global recommendation on physical activity for health for adults is 150 minutes of moderate-intensity activity (or equivalent) per week, measured as a composite of physical activity undertaken across multiple domains: for work (paid and unpaid, including domestic work); for travel (walking and cycling); and for recreation (including sports). For adolescents, the recommendation is 60 minutes of moderate- to vigorous-intensity activity daily.

[u] A medical bone health specialist is defined as any specialist who is caring for BMD deficits in CAYA cancer survivors, such as an endocrinologist (most settings), internist, pediatrician, rheumatologist, family physician, or general practitioner, depending on country and setting.

[v] Defined as fever > 38.3 °C, infective or septic symptoms including hypothermia, hypotension, chills/rigors and changes in mental status (e.g. somnolence, agitation), or animal or human bite with skin break.

[w] Risk of hypothyroidism for all mentioned exposures. Risk of hyperthyroidism after radiotherapy to a volume exposing the thyroid gland, including TBI, or allogeneic HSCT.

[x] MIBG used for diagnostic purposes (e.g. MIBG scanning) does not put patients at risk for hypothyroidism if adequate preventive measures were used.
